# Long-term effects of human amniotic membrane in a rat model of biliary fibrosis

**DOI:** 10.1590/1414-431X20175692

**Published:** 2017-07-03

**Authors:** L.B. Sant'Anna, F.S. Brito, P.R. Barja, M.C. Nicodemo

**Affiliations:** 1 Laboratório de Histologia e Terapia Regenerativa, Instituto de Pesquisa e Desenvolvimento, Universidade do Vale do Paraiba, São José dos Campos, SP, Brasil; 2 Laboratório de Fotoacústica Aplicada aos Sistemas Biológicos, Instituto de Pesquisa e Desenvolvimento, Universidade do Vale do Paraiba, São José dos Campos, SP, Brasil

**Keywords:** Amniotic membrane, Bile duct ligation, Liver fibrosis, Long-term, Quantitative image analysis, Rat

## Abstract

Liver fibrosis is the most common outcome of chronic liver diseases, and its progression to cirrhosis can only be effectively treated with liver transplantation. The amniotic membrane (AM) has been studied as an alternative therapy for fibrosis diseases mainly for its favorable properties, including anti-inflammatory, anti-scaring and immunomodulatory properties. It was recently demonstrated that the AM reduces the progression of biliary fibrosis to its advanced stage, cirrhosis, when applied on the liver for 6 weeks after fibrosis induction. Here, we investigated the effects of AM on rat fibrotic liver, during a prolonged period of time. Fibrosis was induced by bile duct ligation (BDL), and at the same time, a fragment of AM was applied around the liver. After 1, 3, 6, and 9 weeks, the degree of fibrosis was assessed by qualitative Knodell scoring, and by quantitative image analysis to quantify the area of collagen deposition in hepatic tissue. While fibrosis progressed rapidly in untreated BDL animals, leading to cirrhosis within 6 weeks, AM-treated livers showed confined fibrosis at the periportal area with few and thin fibrotic septa, but without cirrhosis. In addition, collagen deposition was reduced to about 36 and 55% of levels observed in BDL at 6 and 9 weeks after BDL, respectively, which shows that the longer the period of AM application, the lower the collagen deposition. These results suggested that AM applied as a patch onto the liver surface for longer periods attenuated the severity of biliary fibrosis and protected against liver degeneration caused by excessive collagen deposition.

## Introduction

Fibrosis is the most common outcome of chronic liver diseases. After repeated injuries, such as viral infection, alcoholism, drug toxicity, autoimmune diseases, and metabolic and biliary disorders (1,2), the liver is subjected to fibrotic remodeling. Fibrosis is characterized by an excessive accumulation of extracellular matrix in hepatic parenchyma, which distorts the normal liver architecture, forming scar tissue that encapsulates the injured area. With disease progression, rings of scar tissue surround regenerative nodules of liver parenchyma, which characterizes the end stage of the disease known as cirrhosis (2). Hepatic cirrhosis is classified among the twenty leading causes of death in the world and the 8th cause of death in Brazil. Patients with cirrhosis have a high risk of irreversible liver failure or hepatocellular carcinoma in adulthood (3). The only effective treatment for cirrhosis is organ transplantation, which still has several limitations, including scarce availability of donor livers, risk of immune rejection, immunosuppressive therapy for life, and the fact that many patients are not able to undergo transplantation (4). Therefore, other therapeutic approaches are needed to serve as alternative for liver transplantation. To this end, human amniotic membrane (AM) represents a potential strategy.

The human AM is the inner part of the fetal membranes, which together with the chorion is discarded after delivery but it could be a useful source of tissues and/or stem cells for transplantation and regenerative medicine (5). The AM-derived cells show great phenotypic plasticity, expressing markers of pluripotent stem cells and are able to differentiate, *in vitro*, toward tissues of all three germ layers (5–7). Furthermore, the AM cells have a low immunogenicity and high immunomodulatory potential, allowing transplantation without acute rejection by the host. According to Parolini et al. (5), this capacity is related to the AM role in maintaining maternal-fetal tolerance, which prevents the mother’s body from rejecting the fetus. Moreover, intact AMs have been widely used in ophthalmology for ocular surface reconstruction (8), repair of burn wounds (9), bone defects (10), and surgical reconstruction of oral cavity (11) and bladder (12). These studies prove that intact AM dressing exert beneficial actions on tissue repair and regeneration due to anti-inflammatory, anti-scaring, analgesic, antibacterial, re-epithelialization, and wound healing effects.

Preclinical studies have shown that xenogeneic and allogeneic transplant of cells derived from the placenta reduced lung fibrosis in bleomycin-treated mice (13), and the use of human AM fragments in the ischemic heart of mice resulted in improved cardiac function (14). Recently, Sant'Anna et al. (15) demonstrated that AM, when applied onto liver, reduced the progression of experimental fibrosis in rats induced by bile duct ligation procedure (BDL). The most acceptable mechanism for the beneficial effects of AM is associated with the release of soluble factors by cells of the AM patch, which exerts paracrine actions on liver tissue (13–15). This decreases the expression of pro-inflammatory and pro-fibrotic cytokines, increasing anti-inflammatory cytokine IL-10 and metalloproteinases, which are proteins that degrade the components of the extracellular matrix (5). However, the anti-inflammatory and antifibrotic mechanisms of AM are still not fully understood, especially in the fibrosis model induced by BDL during a prolonged period of time. Thus, the purpose of this study was to evaluate the effects of human AM when applied to the liver for a long period, on the establishment and progression of fibrosis induced in rats by BDL.

## Material and Methods

### Animals and experimental groups

**Figure 1.**
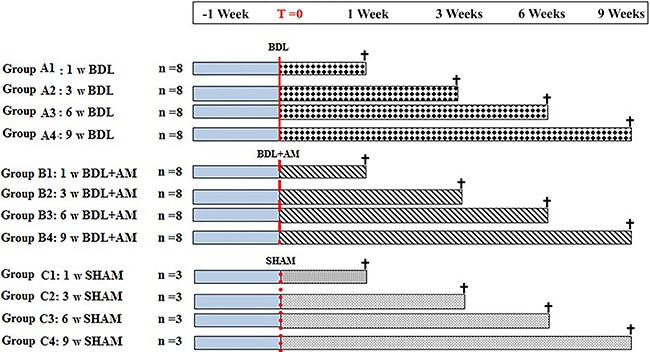
Experimental design. In blue: Period of acclimatization; BDL: bile duct ligation; AM: amniotic membrane.

The study was approved by the Ethics Committee on Animal Use of the Instituto de Pesquisa e Desenvolvimento, Universidade do Vale do Paraiba (protocol No. A12/2013). Seventy-six Wistar rats, weighing approximately 200–250 g provided by Central Animal House of the institution were housed at controlled room temperature (22± 2°C) with daily exposure to a 12:12 h light/dark cycle and unlimited access to food and water. After a week of acclimatization, the animals were randomly divided into groups A, B, and C as shown in Figure 1.

### Collection and processing of amniotic membrane

The study was approved by the Ethics Committee on Human Research of the institution (protocol No. A12/CEP/ 380.403/2013).

**Figure 2.**
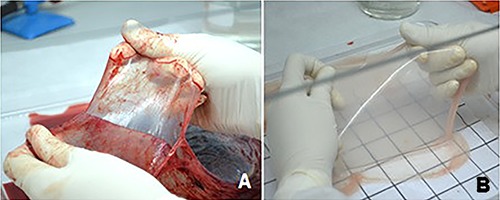
Processing of the amniotic membrane (AM). *A*, Manual separation of AM from chorion; *B*, AM after processing.

Ten human placentas were obtained from elective cesareans of patients with normal pregnancy from the obstetric center of the Municipal Hospital Dr. José de Carvalho Florence in São Jose dos Campos, after prior consent of the mother who signed an informed consent form. Briefly, the AM was manually separated from the chorionic membrane and washed extensively with saline containing 100 U/mL penicillin, 100 mg/mL streptomycin, and amphotericin (Figure 2). The AM was then cut into pieces of suitable size (6×8 cm) with appropriate markings to identify the mesenchymal side of the membrane, and stored separately at room temperature (16) in vials containing 50 mL of DMEM culture medium without added serum and phenol red. Fragments of the AM were applied to the animals within 24 h. As reported by Cargnoni et al. (14), the number and viability of cells in human AM fragments stored for 24 hours are not significantly reduced compared to that observed using fresh membranes.

### Experimental model for the induction of hepatic fibrosis: bile duct ligation

The animals were anesthetized with isoflurane 3% by inhalation, in-camera. Then, each animal was positioned on the operating table and anesthesia was maintained by inhalation of O_2_ and 2.5% isoflurane. Surgical intervention was initiated by shaving and disinfecting the abdominal region, followed by a midline incision and exposure of the common bile duct, which was doubly connected with 4-0 silk suture (Ethicon, Johnson & Johnson, Brazil). The first ligature was in the junction of the hepatic ducts, and the second ligature was made above the entrance of the pancreatic duct. The common bile duct was then transected between ligatures. In the BDL group, the abdominal incision was closed in two layers with 4-0 and 3-0 silk thread sutures (Ethicon, Johnson & Johnson). Thus, the animals had a total permanent biliary obstruction (17).

In the animals of the BDL+AM group, before closure of the abdominal wall, a fragment of AM was added to the liver with its mesenchymal side in contact with the surface of the liver, so that the entire surface of the liver lobes was covered. Holding the upper ends with two clamps, the membrane was inserted below the liver and then moved anteriorly to cover the surface of the medial lobe. The lower ends of the membrane were also raised in order to cover the remaining lobes of the liver. Finally, the upper and lower ends of the membrane were bonded to each other with a bead of methacrylate glue to keep the membrane in place around the liver, avoiding its dispersion into the peritoneal cavity. After this procedure, the abdomen was closed, as described above for the animals of the BDL group. For 5 days after surgery, each animal received antibiotics (Enrofloxacin 0.04 mg/g) and analgesia (Tramadol 0.02 mg/g), both subcutaneously.

After the BDL procedure the rats of both experimental groups, BDL and BDL+AM, were followed up for mortality rate during the 9 weeks of experiment, and for the efficacy of biliary duct obstruction. The rats that did not present dilatation in the diameter of the bile duct, absence of ductular reaction (i.e., increased number of ductules and ducts), fibrosis in the liver tissue, and those that died during the course of the experiment were excluded and replaced by other animals.

### Euthanasia of animals, collection of biological samples, and histologic processing

One, 3, 6, and 9 weeks after the BDL surgery, the animals of all groups were anesthetized to remove the liver. The specimens were fixed in 10% buffered formalin for 48 h. They were then embedded in Paraplast (Sigma-Aldrich, Germany) and sectioned with an automatic microtome to obtain 4-μm thick histological sections. The sections were stained with Masson's trichrome and Sirius Red, for qualitative histological analysis of the degree of fibrosis and quantitative image analysis, respectively. At the end of the collection, the animals were euthanized with an overdose of isoflurane.

### Qualitative analysis of fibrosis degree

The degree of liver fibrosis was assessed qualitatively using an optical microscope (Olympus BX-41) at 100× by applying the scoring system for fibrosis (category IV) of Knodell, as follows (18): score 0: no fibrosis; score 1: fibrous portal expansion; score 3: bridging fibrosis (portal-portal or portal-central); score 4: cirrhosis. This analysis was performed by a pathologist who was not aware of the treatment of rats (blind study). The average score of 10 random fields per histologic section from each rat liver was used to create a single score for each specimen in each experimental group.

### Quantitative image analysis

The slides stained by Sirius Red were quantitatively assessed by digital image analysis to obtain the area occupied by collagen deposition in liver tissue. Quantitation was performed using the automated image analysis open-source CellProfiler software (Broad Institute of Harvard and MIT, USA). Microscopic images were captured by a digital video camera (Leica DF425, Germany) coupled to an optical microscope (Leica DM2500) and scanned at 1024×768 pixels, 24 bits/pixel resolution with an overall magnification of 100×. Digital images were processed by CellProfiler (Broad Institute of Harvard and MIT), which automatically identified, isolated, and measured the areas occupied by collagen (red) relative to the total image area. The mean percentage of wound area in 10 histological fields centered on the central vein and chosen randomly was used to generate a unique value for each sample in each experimental group.

### Statistical analysis

Score and collagen data are reported as medians and interquartile range (IQR) and represented in boxplot graphs (with comparison between groups, for each week). Scores and collagen data are also reported as means and standard deviation and plotted in Line Graph with Origin 7.5 (OriginLab, USA) in time evolution analysis, that is, comparing the same group over the weeks. Unpaired *t*-test was performed to evaluate the statistical significance of the difference between experimental groups in each treatment week. To evaluate the significance between different weeks in the same experimental group, we used the single factor ANOVA followed by Tukey's multiple comparison test. We used Fisher's exact test to compare the mortality rates. A value of P<0.05 was considered statistically significant (*) and the value of P<0.01 (**), very significant. Data analysis was performed with Instat 3.0 software (GraphPad, USA) and the construction of graphs with Origin 7.5 (OriginLab) and BioEstat 5.3 (Mamirauá, Brazil) programs.

## Results

### General aspects and mortality rate

At the end of the different experimental times (weeks 1, 3, 6, and 9), the efficacy of biliary duct obstruction in inducing fibrosis was assessed by the observation of bile duct dilatation and histopathological features specific of biliary fibrosis. The macroscopic analysis demonstrated bile duct dilatation in different intensities regarding the presence of cyst at porta hepatis with a greenish or yellow-whitish fluid. The longer the ligation time, the greater the dilatation of the duct. Considering all rats, BDL (n=32) and BDL+AM (n=32), we observed the absence of bile duct dilatation in 7 rats (10.93%), 4 in the BDL group (12.5%) and 3 in the BDL+AM (9.3%). The histological analysis of liver tissue of these rats demonstrated absence of ductular reaction and subsequent periductular fibrosis. These rats were replaced by other rats that presented bile duct dilatation and ductular reaction.

The mortality rate during the 9 weeks of experiment was 17.18% (11 rats). In BDL group (n=32) 8 rats died (25%), and in BDL+AM group (n=32), only 3 rats died (9.37%) without significant difference between groups at the end of 9 weeks after fibrosis induction (P>0.05; Fisher's exact test). In BDL group the majority of deaths occurred in the latest 2 weeks after ligation (week 1: 1 death; week 3: 1 death; week 6: 3 deaths; week 9: 3 deaths). In contrast, in BDL+AM rats the mortality rate was lower at all time points (week 1: 1 death; week 3: 0 death; week 6: 1 death; week 9: 1 death). However, no significant differences were found between groups at each time point.

### Qualitative analysis

**Figure 3.**
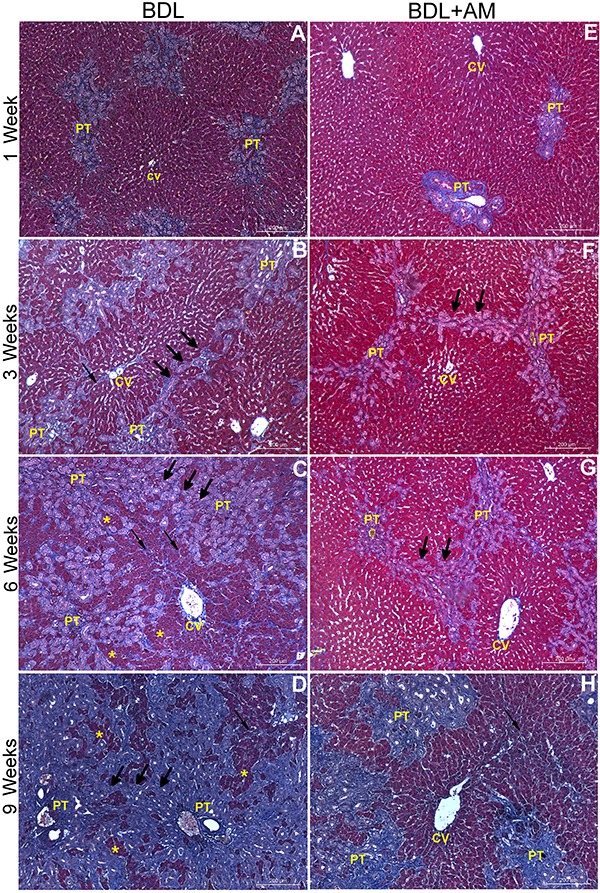
Representative microphotographs of liver fibrosis progression, in the experimental groups BDL (A-D) and BDL+AM (E-H), at 1, 3, 6, and 9 weeks after fibrosis induction. BDL: bile duct ligation; AM: amniotic membrane; CV: central vein; PT: portal tract with ductular expansion; thick arrows: connection (fibrotic septa) between the expanded portal tracts; thin arrows: interstitial collagen fibers in the hepatic parenchyma; *: nodules of hepatocytes. Masson's trichrome staining with magnification of 100×.

**Figure 4.**
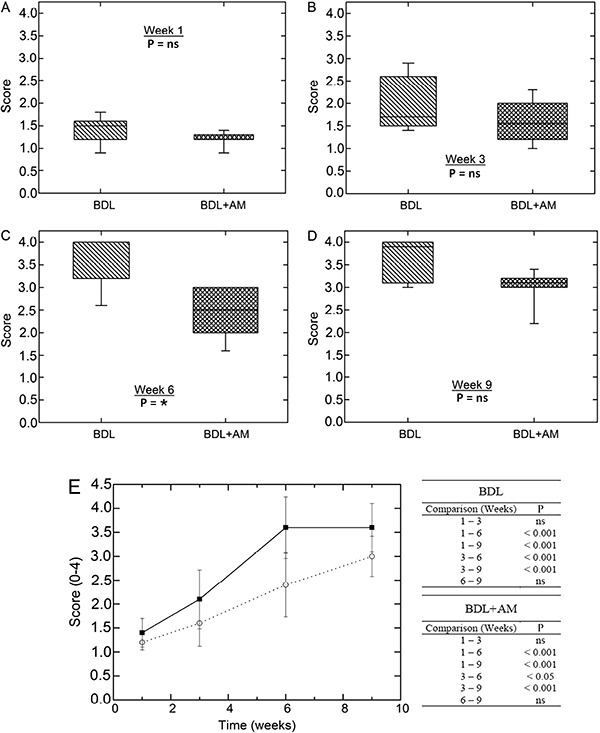
Qualitative evaluation of fibrosis degree by Knodell scoring. Data are reported as median and interquartile range of the fibrosis score in the experimental groups, BDL and BDL+AM at week 1 (*A*), week 3 (*B*), week 6 (*C*), and week 9 (*D*), after fibrosis induction. *P<0.05, unpaired Student's *t*-test. ns: not significant. *E*, Means±SD of fibrosis score progression in BDL (squares) or BDL+AM (circles), at different time points. The tables represent the significance between time points in BDL and BDL+AM groups (one way ANOVA followed by Tukey's multiple comparison test. BDL: bile duct ligation; AM: amniotic membrane.

Qualitative analysis was done first, due to its importance in characterizing the stage of fibrosis, based on changes in the liver tissue architecture. The results of these analyzes are shown in Figures 3 and 4. Figure 3 shows photomicrographs of liver fibrosis in both experimental groups after 1, 3, 6, and 9 weeks. In the first week, fibrosis was confined in the periductular region along the portal tracts enlarged by ductular expansion within the liver parenchyma (Figure 3A and E). Subsequently, at 3 weeks, the fibrous portal expansion infiltrated the liver parenchyma. Both groups (Figure 3B and F) showed connections between the portal tracts or fibrotic septa ("bridging fibrosis"). In the BDL group, in addition to more "bridges" connecting the portal spaces, there was also the formation of connections between portal areas and the central vein, characterizing a more advanced degree of the fibrosis. Additionally, at 3 weeks, the formation of collagen fibers started in the hepatic parenchyma (interstitial collagen), between hepatocytes, and sinusoidal.

At 6 weeks, the architecture of the liver was seriously compromised in the BDL group (Figure 3C), with an increase in the number of fibrotic septa, an intense interstitial collagen in the parenchyma, and even an extensive ductular expansion circumscribing nodules of hepatocytes in the liver parenchyma, which are characteristic of cirrhosis. In contrast, in the BDL+AM group (Figure 3G), the architecture of hepatic tissue was more preserved. The fibrosis remained confined to enlarged portal tracts, and the ductular expansion and fibrotic septa were lower compared to the BDL group, and there was no formation of nodules of hepatocytes.

At 9 weeks in the BDL group (Figure 3D), the cirrhosis progressively worsened, with an excessive ductular expansion not only in portal tracts, but also in the entire liver parenchyma, delineating multiple nodules of hepatocytes. In the BDL+AM group (Figure 3H), damage in hepatic tissue was significantly lower compared to the BDL group. The liver architecture was more preserved in fine fibrotic septa and without presenting extensive connections between the portal tracts.

The results of the qualitative evaluation of fibrosis degree by the Knodell scoring system are reported in Figure 4. In the first and third week (Figure 4A and B), the fibrosis scores in the BDL+AM group were lower than in BDL group (1.2±0.16 *vs* 1.4±0.3 and 1.6±0.48 *vs* 2.1± 0.62, respectively), but without a significant difference (P>0.05). In contrast, at 6 weeks, the AM-treated group had a significantly lower score than the BDL group (2.4± 0.67 *vs* 3.6±0.64; P<0.05; Figure 4C). As in the first and third weeks, no statistically significant difference between experimental groups was found after 9 weeks, although the highest score was observed in the BDL group compared to the BDL+AM group, (3.6±0.5 *vs* 3.0±0.4; P>0.05).

Regarding the temporal progression of liver fibrosis with groups, the results showed no significant difference in disease progression between the first and third week (P>0.05), i.e., the value of fibrosis score remained similar in the BDL group (week 1: 1.4±0:30 *vs* week 3: 2.1± 0.62; P>0.05) and in the BDL+AM group, (week 1: 1.2± 0:16 *vs* week 3: 1.6±0:48; P>0.05) (Figure 4). In contrast, between the third and sixth weeks, the fibrosis score in the BDL group showed rapid progression and was extremely significant (week 3: 2.1±0.62 *vs* week 6: 3.6± 0.64; P<0.001) compared to the AM group, in which the score progression was slow and less significant (week 3: 1.6±0:48 *vs* week 6: 2.4±0.67; P<0.05). In the sixth and ninth week, both groups had no significant increase despite the group treated with AM having a lower score compared to the BDL group.

### Quantitative analysis

**Figure 5.**
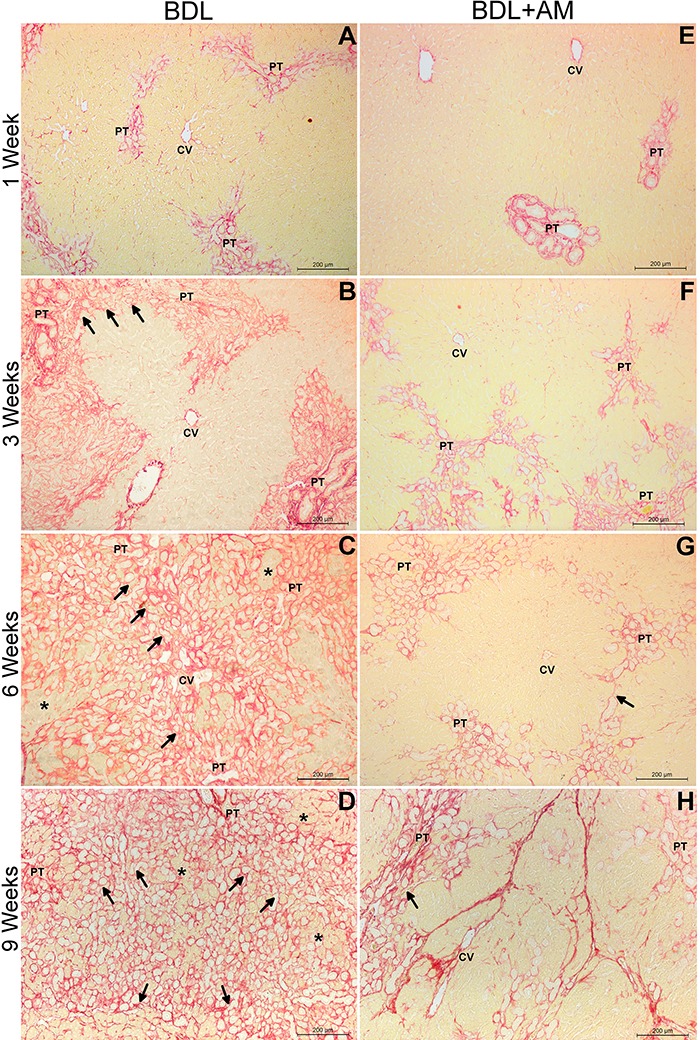
Representative microphotographs of liver fibrosis progression in the experimental groups BDL (A–D) and BDL+AM (E–H), at 1, 3, 6, and 9 weeks after fibrosis induction. BDL: bile duct ligation; AM: amniotic membrane; CV: central vein; PT: portal tract with ductular expansion; arrows: connection (fibrotic septa) between the expanded portal tracts; *: nodules of hepatocytes. Sirius Red staining with a magnification of 100×.

**Figure 6.**
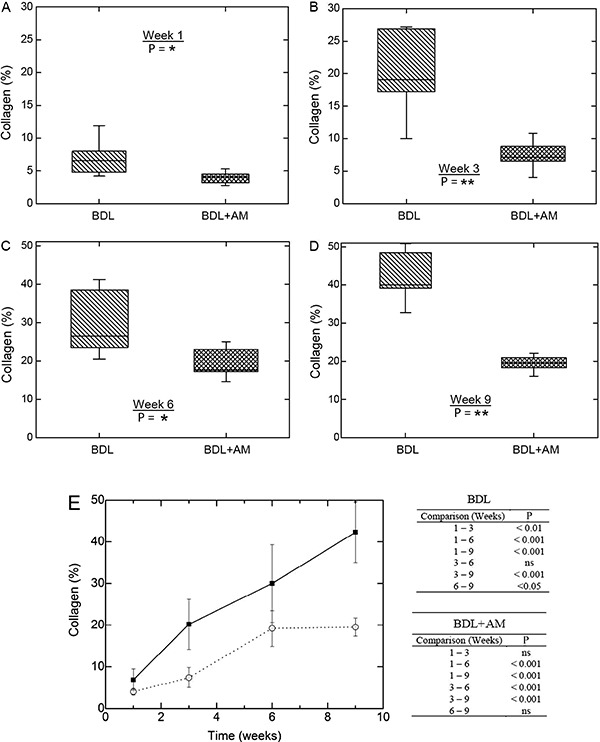
Quantitative evaluation of collagen deposition. Median with interquatile range of collagen deposition in the experimental groups BDL and BDL+AM at week 1 (*A*), week 3 (*B*), week 6 (*C*), and week 9 (*D*) after fibrosis induction. *P<0.05; **P<0.01, unpaired Student's *t*-test. *E*, Means±SD of collagen deposition progression in BDL (squares) or BDL+AM (circles) groups at different time points. The tables represent the significance between time points in BDL and BDL+AM groups, respectively (one way ANOVA followed by Tukey's multiple comparisons test, ns: not significant). BDL: bile duct ligation; AM: amniotic membrane.

Representative photomicrographs and quantitative data after 1, 3, 6, and 9 weeks are shown in Figures 5 and 6, respectively.

**Figure 7.**
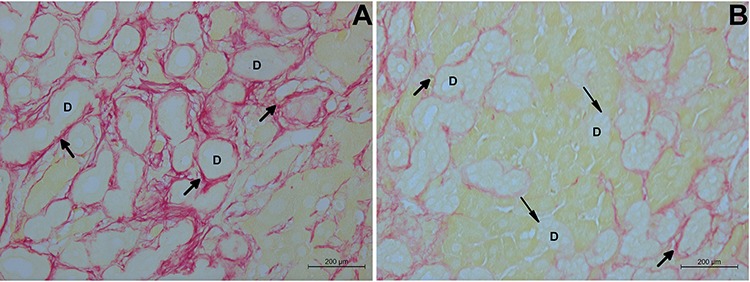
Representative microphotographs of liver with biliary fibrosis in the experimental groups, BDL (*A*) and BDL+AM (*B*), 9 weeks after fibrosis induction. D: biliary structures; thick arrows: collagen fibers in the periductular region; thin arrows: absence of collagen fibers in the periductular region. Sirius Red staining, magnification 400×. BDL: bile duct ligation; AM: amniotic membrane.

At all experimental times, statistically significant differences were observed between the BDL and BDL+AM groups in collagen deposition. At 1 week, the percentage of collagen deposition in the BDL+AM group was lower (4.1%) compared to BDL group (6.6%). The collagen accumulated in periductular region along the expanded portal tracts and in the hepatic parenchyma which was not observed in the group with AM. At 3 weeks, the BDL group had a significant increase (P<0.01) in area occupied by collagen (19.1%) compared to the BDL+AM group (7.1%, P>0.05). At 6 weeks, in the BDL+AM group, the area occupied by collagen in the liver (17.5%) was also lower than the BDL group (26.4%). In the same group, at 9 weeks, the total liver architecture was impaired with the significant accumulation of collagen (40.0%) in periductular regions, which formed thick fibrotic septa that connected all expanded portal tracts and the central vein, isolating hepatocytes groups and forming the cirrhotic nodules in the liver parenchyma. In the BDL+AM group, the amount of collagen deposition was significantly lower than the BDL group, (19.7 *vs* 40.0%, P<0.01), and remained stable, with the same amount of collagen observed in the sixth week of the same group (17.5%). In addition, in the BDL+AM group not all biliary structures were involved by periductular collagen (Figure 7).

Regarding the temporal progression of collagen deposition in the same group, the progression of the area occupied by collagen was considerably greater in the BDL group (week 1: 6.9% *vs* week 9: 42.3%, P<0.001) than in the BDL+AM group (week 1: 4.0% *vs* week 9: 19.5%; P<0.001, Figure 6E). In the latter group, the progression of the area occupied by collagen was only 4.01% in the first week and 7.41% in the third week, with the differences not statistically significant. However, in the BDL group the area occupied by collagen progressed from 6.9 to 20.2% in the same experimental times, with a very significant difference (P<0.01). In addition, at 6 weeks, the area occupied by collagen deposition reached 19.2% of the liver tissue in BDL+AM group, while in the BDL group, it progressed rapidly to 30% at 6 weeks and to 42% at 9 weeks (P<0.05). In contrast, in the BDL+AM group the collagen deposition area remained at 19%, from week 6 to week 9.

## Discussion

The objective of this study was to evaluate the potential of AM on the biliary fibrosis when applied to the surface of the liver at the same time of the fibrotic stimulus and maintained for a 9-week period. The main result of our study was that the prolonged application of AM onto liver reduced and stabilized the area occupied by collagen deposition in the hepatic parenchyma caused by fibrotic degeneration.

No animal model recapitulates completely all the pathophysiological aspects of human liver fibrosis (19). However, BDL is a common experimental model widely accepted and used in rats, which reproduces and allows the study of the biliary fibrosis that originates from several chronic liver diseases caused by cholestasis, including biliary atresia (1). Fibrosis induced by BDL model involves three main processes: ductular reaction, which mainly refer to a high number of bile ducts and ductules (biliary structures) (20); cellular differentiation of portal fibroblast and/or hepatic stellate cells into myofibroblasts, which are responsible for the unbalanced collagen synthesis; and consequently, the excessive deposition of collagen in the matrix (21).

Despite technological advances in recent decades and the emergence of advanced image and molecular biology, the diagnosis of fibrosis still depends on the histopathological examination of liver biopsy (22,23).

In our study, the results of qualitative histological analysis, which takes into account the pathological changes in the liver tissue visualized with Masson's trichrome method, showed that in the BDL group, fibrosis initiated in week 1, with portal expansion (Knodell score1), and progressed to the intermediate stage in the third week, with the presence of fibrotic septa connecting portal spaces. Between the third and sixth weeks, fibrosis progressed quickly, with significant increases from one period to the next, reaching the advanced stage of cirrhosis in 6 weeks after the BDL, which was maintained in the ninth week. These data confirm that the BDL model was efficient to induce biliary fibrosis, and is, therefore, in accordance with other studies (24–26), which consider the early stage of fibrosis the period between the first and second week, the intermediate stage between the third and fourth weeks, and the advanced stage beginning in the fifth week after BDL. Rats treated with AM also showed fibrosis at the early stage in the first week, and intermediate fibrosis in the third week, however, with lower values than the BDL group. Moreover, the BDL+AM group demonstrated a slower evolution between the third and the sixth week, and even slower in the period between the sixth and the ninth weeks, not reaching the stage of cirrhosis. In contrast, in the BDL group, cirrhosis was already observed in the sixth week after BDL indicating that the AM slows fibrosis progression. Our results were similar to those of Sant'Anna et al. (15) who observed no significant difference in the severity of fibrosis in the early period, comparing the groups with and without AM, after 2 weeks of fibrotic stimulus. However, after 4 weeks from the AM application, fibrosis began to mitigate, and was significantly reduced after six weeks from BDL. In addition, Ricci et al. (27), demonstrated the potential of both fresh and cryopreserved AM in reducing fibrosis and preventing its progression to cirrhosis. Thus, to exert a beneficial effect on biliary fibrosis, AM patching must remain in the liver for at least 4 weeks.

To our knowledge, our work is the first to evaluate qualitatively and quantitatively the potential of AM on the fibrosis up to the ninth week. Previous studies (15,27) only evaluated the effect of AM up to 6 weeks from BDL, which motivated us to keep the AM in contact with the fibrotic liver for a longer period. At 9 weeks, the qualitative analysis by Knodell scoring system demonstrated a lower fibrosis score in livers treated with AM, but without significant difference compared to the BDL group. Although histopathological information is recognized as the most valuable data for fibrosis assessment, conventional histology categorical systems describe the changes of fibrosis patterns in liver tissue, but the simplified ordinal digits assigned by these systems cannot reflect the fibrosis dynamics with sufficient precision and reproducibility (28). Additionally, other authors (23,29,30) demonstrated that although visual analysis of fibrosis is acceptable for a few samples, the observation of many samples is time consuming, subjective, and cannot be quantified. To overcome the limitation of being semi-quantitative or qualitative, researchers have been trying to fully quantify fibrosis in liver biopsy based on the strategy of morphometric assessment, which includes fibrous collagen-specific staining, digital imaging of the tissue section, and computer assisted digital image analysis (28,31). Thus, in our work, for quantifying collagen deposition, liver samples were stained with Sirius Red, and, as in previous studies by Sant'Anna et al. (15,32), the quantitative analysis of collagen was performed using the image analysis software CellProfiler.

The results of the quantitative analysis demonstrated a significant reduction in the percentage of collagen in the liver tissue when treated with AM in all experimental periods, especially at 9 weeks after its application, with about 50% reduction of levels observed in BDL group. Even though the qualitative analysis did not show a significant difference between fibrosis scores at 9 weeks, the reduced collagen deposition in liver tissue is a very important finding, because the quantitative analysis is more specific and objective than qualitative analysis, which may have been influenced by the number of samples, biopsy size, intra-observer, and inter-observer variations (23,29,30). Morphometric assessment by computer-assisted digital image analysis detects changes in fibrosis amount (i.e., collagen) as a continuous variable, and has shown its independent diagnostic value for assessment of advanced or late-stage fibrosis and especially for diagnosis of disease regression (28).

When measured qualitatively, the fibrosis of the BDL group reached its maximum degree known as cirrhosis (Knodell score 4) in the sixth week and remained in the ninth week. However, when measured quantitatively, the collagen content at 9 weeks was greater than at 6 weeks, indicating that the same score or degree of fibrosis may have different collagen content. According to Standish et al. (22), this is because the qualitative analysis considers the cirrhosis stage only by the presence of fibrotic septa surrounding parenchyma nodules, without taking into account the thickness of these fibrotic septa, which varies according to the amount of collagen deposition. Indeed, Wang and Hou (28,33) postulated that the qualitative analysis by scoring systems alone is not enough to correctly evaluate fibrosis in advanced stages because categorical systems simply classify cirrhosis into one or two stages and do not adequately reflect the complexity of the cirrhotic condition, especially concerning the amount of fibrillar collagen, which is a major component underlying the architecture of hepatic fibrosis. Taken together, our findings show that when there is no treatment, the level of cirrhosis is reached and, even if it is maintained in the long-term, the collagen content increases, thus inducing a great hepatic parenchyma degeneration that can compromise liver functions. In contrast, when livers are treated with AM patches the collagen deposition is less and can stabilize with time.

Considering the importance of collagen for fibrosis, another important finding of our work was the absence of homogeneous distribution of collagen around all biliary structures, in the BDL+AM group at 9 weeks. There were periductular regions with little collagen thickness, and in some samples the collagen was almost absent. According Beaussier et al. (34), in biliary fibrosis, the resident fibroblasts of portal tracts in the connective tissue around the vessels and biliary structures are the main cells responsible for differentiating into myofibroblasts, which once activated, proliferate, migrate intensely, and secrete collagen that accumulate in the portal and periductular regions. Thus, the periductular region is composed of one or more processes of myofibroblasts that after activation acquire a stellate shape due to the presence of actin microfilaments in the cell cytoskeleton, allowing its identification by the immunohistochemical expression of the protein α-smooth muscle actin (α-SMA) (35).

Sant'Anna et al. (15) demonstrated that the application of fresh human AM around the fibrotic liver decreases the immunoreactivity of α-SMA in all the studied time points compared with the BDL group. Moreover, at 4 to 6 weeks, they observed a significant reduction in the number of myofibroblasts. Furthermore, Ricci et al. (27) found that in the group with fresh or cryopreserved AM not all cells around the biliary structures were immunopositive for α-SMA, and considered the result to be strong evidence of the AM action in the induction of apoptosis or reversal of myofibroblast phenotype. Although our study did not evaluate the myofibroblasts, based on these previous studies, the reduction in collagen deposition, including periductular regions that were not homogeneously stained by Sirius Red, was due to the action of AM on myofibroblasts.

In biliary fibrosis, the ductular reaction or abnormal expansion of biliary structures is the first pathophysiological change that occurs after bile duct obstruction, thus playing an important role in the initiation and progression of the disease (1,21,20). Our qualitative analysis demonstrated that, in the BDL group, ductular reaction was extensive at 6 weeks and even higher at 9 weeks, as well as collagen deposition evaluated by quantitative analysis. On the contrary, in the group treated with AM, both the ductular reaction and collagen deposition were lower compared to the BDL group. These data show that fibrosis developed close to the ductular reaction, which is consistent with Beaussier et al. (34), Chen et al. (17), and Sant'Anna et al. (15) and suggests that the ductular reaction may be one of the targets of AM on the reduction of the biliary fibrosis.

The exact mechanism of the effect of AM on the reduction of fibrosis specifically induced by BDL is not yet fully elucidated, and up to now we can only speculate about such mechanisms. According to Manuelpillai et al. (7), the most likely mechanism is associated with the release of soluble factors secreted by the AM cells in paracrine action, which act on the hepatic tissue, thus reducing the expression of pro-inflammatory and pro-fibrotic cytokines (TGF-β, PDGF, IL-6), and increasing IL-10 anti-inflammatory cytokines and metalloproteinases, which are proteins that degrade the extracellular matrix of the tissue. Such mechanism based on paracrine action, rather than the action of cells in the tissue, was hypothesized by Tsai et al. (36) to explain the observed reduction of liver fibrosis induced by CCl4 after the use of mesenchymal stem cells, because that study found no stem cells inside the liver. Additionally, Ricci et al. (27) comparing the potential of fresh and cryopreserved AM, demonstrated that even with low cell vitality, cryopreserved AM showed anti-fibrotic effects similar to those of fresh human AM. Thus, these studies support the notion that human AM appears to act primarily as a matrix and as a source of bioactive factors (7). The results of these studies justify the reason for applying the AM with the mesenchymal side facing the surface of the liver, in the present study.

Although all the soluble factors released from AM are not yet known, one of the targets of the paracrine action of these factors could be the TGF-β, which is considered the main fibrogenic factor for the progression of fibrosis, acting directly in the synthesis and secretion of excessive components of the extracellular matrix (37). The reduction in the amount of TGF-β in the hepatic tissue has been used to explain part of the mechanism of fibrosis reduction observed in other therapies such as administration of curcumin (38), chromium (17), and artemisia herb (39). Indeed, the recent study published by Sant'Anna et al. (40) confirmed that AM applied to the liver after 2 weeks of BDL reduces the expression of TGF-β to about 62% compared to untreated controls.

Taken together, the results of qualitative analysis of the degree of fibrosis and quantitative morphometric analysis of collagen deposition suggested that human AM might serve as a protective agent against liver tissue damage associated with fibrotic degeneration, perhaps being a strategy to delay and/or reduce the need for liver transplants in some cases. However, before the AM can be used as an effective approach to fibrotic diseases in clinical settings, further preclinical studies are needed to explore other targets of the anti-fibrotic effects of human AM patching in fibrosis induced in the BDL model. In conclusion, our study demonstrated that the AM, when applied onto liver surface immediately after BDL stimulus and for the period of 9 weeks, did not prevent the establishment of biliary fibrosis, but it certainly reduced the severity of fibrosis and stabilized the collagen deposition in liver tissue, thus reducing the progression of biliary fibrosis to the end stage of disease.
